# Late *Campylobacter jejuni* mastitis after augmentation mammoplasty

**DOI:** 10.1016/j.jpra.2021.06.004

**Published:** 2021-06-18

**Authors:** Tomas Vedin, Henrik Bergenfeldt

**Affiliations:** a Department of Clinical Sciences, Faculty of Medicine, Lund University, Lund, Sweden; b Department of Surgery, Helsingborg Hospital, Helsingborg, Sweden

**Keywords:** Augmentation mammoplasty, Breast-Implant associated infections, Campylobacter

## Abstract

Breast implant-associated infections (BIAI) occur in approximately 2% of patients after augmentation mammoplasty. In some cases, BIAI can be treated conservatively, whereas others need implant removal. Knowledge of uncommon potential pathogens in BIAI is important to ensure optimal treatment of BIAI. In the present case report, we describe a case of bilateral late *Campylobacter jejuni* mastitis in a 34-year-old woman without previous symptoms of gastroenteritis. While *Staphylococci* are common causative pathogens in BIAI, there are numerous potential pathogens. This case highlights the importance of careful consideration of antibiotic treatment and switch to broad-spectrum antibiotic regimen in BIAI not responding to initial treatment.

## Introduction

Cosmetic augmentation mammoplasty using artificial implants is a commonly performed procedure, with approximately 300,000 procedures performed annually in the U.S.^1^. Although serious complications after the procedure are rare, breast implant-associated infections (BIAI) occur in approximately 2% of cases^2^.

BIAI are culture-positive in approximately 75% of cases. *Staphylococcus* species (coagulase negative *Staphylococci* and *S. Aureus*) are the most common culprit organisms representing 50% of cases. The second most common pathogen species is *Pseudomonas* followed by *Peptostreptococcus*. As shown in Box 1, a wide variety of pathogens have been found to cause BIAI^3^^,^^4^.

**Box 1:** Previously reported causative pathogens in BIAI.^2^, ^3^, ^4^

Gram-positive	Gram-negative
*Staphylococcus Aureus*	*Pseudomonas*
Coagulase-negative *Staphylococci*	*Serratia*
*Streptococci*	*Enterobacter*
*Enterococci*	*Acinetobacter*
*Propionibacterium*	*Proteus*
*Peptostreptococci*	*Escherichia Coli*
*Corynebacterium*	*Klebsiella*
*Peptoniphilus*	*Morganella*
*Arcanobacterium*	*Stenotrophomonas*
*Bacillus*	*Citrobacter*
*Kocuria*	*Bacteroides*
Diphtheroid	*Xanthomonas*
	*Pasteurella*
**Mycobacteria**	

The management of BIAI varies considerably with explantation rates ranging from 25% to 95%, depending on indication and severity of infection^4^. Another possible cause of variability in treatment results could be uncommon and therefore, unsuspected causative microorganisms. If the causative pathogen is not considered when deciding on antibiotic regimen, this could result in inadequate antibiotic coverage. Knowledge of uncommon potential pathogens in BIAI is therefore important to ensure optimal treatment of BIAI. In the present case report, we describe a case of bilateral late *Campylobacter jejuni* mastitis. The patient consented to the writing of this report.

## Case report

A 34-year-old woman presented with a 2-day history of pain in the right breast. She had undergone bilateral cosmetic supramuscular augmentation mammoplasty 17 years ago but was otherwise healthy. She denied symptoms of diarrhea, vomiting, or abdominal pain in the last year. However, 2 months prior to this event, she had ceased breastfeeding. She was tachycardic and had a fever of 39°C. On examination, she was found to have bilateral capsular contractures and a diffuse erythema along the caudal lateral border of the right breast. Mammogram and sonogram of the breasts were obtained and indicated inflammatory reaction immediately lateral to the right breast implant. Mammography also showed a capsular rupture of the left implant. The patient was diagnosed with breast implant-associated mastitis and was started on oral flucloxacillin, mainly targeting *Staphylococcus* species.

Five days later, the patient returned to the emergency department due to a sudden worsening of symptoms. She complained of dizziness and fatigue. She had a pulse of 115 beats per minute, a systolic blood pressure of 90 mmHg, and a temperature of 38.1°C. The laboratory workup showed a white blood cell (WBC) count of 14.1 × 10^9^/L and a C-reactive protein (CRP) of 152 mg/L. The patient was admitted to the surgical ward and switched to intravenous flucloxacillin after consultation with an infectious disease specialist. A repeat sonogram rendered similar findings as the initial sonogram did, i.e., minimal amounts of free fluid and inflammatory reaction of adjacent tissue.

The patient was treated with intravenous flucloxacillin for 3 days with worsening of the clinical condition. Because of increasing tenderness and erythema of the right breast and increasing CRP and WBC, during antibiotic treatment, the patient was recommended removal of the implants.

Eight days after the initial presentation, implants were surgically removed. During surgery, capsular rupture of the left implant was confirmed. There was pus in the prosthetic cavity, and a culture was therefore obtained. The right breast implant was intact. However, on the dorsal aspect of the prosthetic cavity, there was fibrosis of the breast implant capsule. The fibrotic capsular tissue was sent for examination by a pathologist, and cultures were obtained from both surgical sites. Bilateral drains were placed and antibiotics were continued. The patient was discharged 2 days postoperatively. Cultures from both breasts grew *C. jejuni*. After the isolation of *Campylobacter,* antibiotics were switched to ciprofloxacin as the strain was susceptible to this antibiotic. Drains were removed in the outpatient clinic.

## Discussion

In the present case report, we describe a case of breast implant-associated bilateral mastitis with *C. jejuni* as the causative organism occurring 17 years after the original implantation.

While BIAI is a well-known entity, to our knowledge, the present case report is the first description of *Campylobacter* species as the causative organism. However, *Campylobacter* has infrequently been reported as the causative organism in other foreign body infections such as prosthetic joint infections^2^^,^^5^. It is, therefore, feasible that *Campylobacter* can be the causative organism in BIAIs.

BIAIs occurring late after implantation are generally believed to be due to hematogenous seeding to the implant pocket during bacteremia^2^. *Campylobacter* has been shown to cause such bacteremia, although it is a rare occurrence with an annual incidence of laboratory-verified bacteremia of 0.3/100,000 cases^6^. While *Campylobacter* bacteremia is rare, a study of all cases of blood culture-positive *Campylobacter* infections in Finland found that approximately 20% of patients had *Campylobacter* bacteremia without gastrointestinal symptoms^6^. It would therefore seem that this is the most likely route of infection in the patient described in this case report. Regrettably, no stool cultures were obtained from the patient.

Another possible mode of infection is contamination with *Campylobacter* bacteria during the initial breast augmentation surgery. However, as this would mean a colonization or subclinical infection of the breast implants for 17 years before the emergence of an overt infection, this route of infection seems highly unlikely.

The patient in the present case report was treated with flucloxacillin and breast implants were removed after failing antibiotic therapy. *Campylobacter* species are not generally susceptible to flucloxacillin^7^. Thus, although an infectious disease specialist was consulted, the patient did not initially receive antibiotics targeting the causative pathogen. It remains unknown if administration of the correct antibiotic would have resulted in successful treatment of the infection and salvage of the implants. Notwithstanding the antibiotic regimen, the patient would have been recommended removal of the prostheses because of the left-sided capsular rupture. However, given the rather high rate of causative organisms not susceptible to flucloxacillin in previous studies, we advocate a switch to a broad-spectrum antibiotic as the first measure in patients failing initial antibiotic treatment. Furthermore, given the high frequency of coagulase-negative *Staphylococci,* addition of vancomycin should also be considered as coagulase-negative *Staphylococci* are frequently methicillin-resistant^8^. Ideally treatment should be directed to the causative organism rather than empiric treatment. We did not attempt to obtain material for culture prior to surgical removal of the implants but believe that ultrasound-guided aspiration of peri-implant fluid as recommended by Pittet et al is advisable if possible^2^.

After cultures grew *Campylobacter,* the patient was switched to ciprofloxacin as antimicrobial susceptibility testing showed that the cultured strain was susceptible to ciprofloxacin. However, this particular antibiotic is generally not recommended for treatment of *Campylobacter* due to a high risk of antibiotic resistance^9^. Therefore, we do not advocate this as the general empiric treatment in suspected breast implant-associated *Campylobacter* infections. The current recommendation for treatment of infectious diarrhea caused by *Campylobacter* is azithromycin, which we believe would therefore be a natural first choice of antibiotic treatment in *Campylobacter*-associated BIAI^10^.

In conclusion, *C. jejuni* is a possible causative pathogen in late BIAI. The prognosis and route of infection in such infections is largely unknown. This case highlights the importance of careful consideration of antibiotic treatment and switch to a broad-spectrum antibiotic regimen in BIAI not responding to initial treatment.

## Declaration of Competing Interest

N/A.
